# Evaluation of crude watermelon oil as lubricant in cylindrical turning of AISI 1525 steel employing Taguchi and grey relational analyses techniques

**DOI:** 10.1016/j.heliyon.2024.e25349

**Published:** 2024-02-01

**Authors:** R.A. Kazeem, D.A. Fadare, I.G. Akande, T-C. Jen, S.A. Akinlabi, E.T. Akinlabi

**Affiliations:** a Department of Mechanical Engineering, University of Ibadan, Ibadan, 200005, Nigeria; b Department of Mechanical Engineering Science, University of Johannesburg, Auckland Park, Johannesburg, 2006, South Africa; c Department of Automotive Engineering, University of Ibadan, Ibadan, 200005, Nigeria; d Department of Mechanical and Construction Engineering, Faculty of Engineering and Environment, Northumbria University, Newcastle, NE7 7XA, United Kingdom

**Keywords:** Watermelon oil, Mineral oil, Lubricants, Grey relational analysis, AISI 1525 steel

## Abstract

Cutting fluids are used for cooling and lubricating the machining area of components used in manufacturing industries such as aerospace, automotive, petroleum, and heavy machinery. Mineral oils derived from petroleum are commonly utilized as cutting fluids. Mineral oil is hazardous to the health of workers and damaging to the environment. There is a need for a substitute for mineral oil. Vegetable oil is increasingly being used as a cutting fluid. Vegetable oils are easily accessible and have benefits including excellent biodegradability, resistance to fire, low humidity rates, and a low coefficient of expansion under heat. This study adopts watermelon oil as a lubricant in MQL machining of AISI 1525 steel using tungsten tools. In the experiment, the feed rate, depth of cut (DC) and spindle speed were varied using the Taguchi L_9_ orthogonal array. Grey relational analysis was conducted to obtain optimum cutting parameters for surface roughness, machine vibration, and cutting temperature. Hardness and microstructural analysis of the workpiece were also conducted. Results showed that vegetable oil performed much more effectively than mineral oil in most experiments. The DC was shown to be the most efficient cutting parameter after applying ANOVA analysis based on the parameters that were evaluated. Additionally, models for cutting temperature, machine vibration, and surface roughness values have been developed with accuracy between 69.73 % and 99.05 %. The hardness of the workpiece increases with an increase in diameter, which was attributed to the increase in the steel rod (workpiece) cross-sectional area and the likelihood of a more uniform stress distribution. Moreover, finer grain sizes were observed at 70 mm diameter, with the predominant presence of pearlites. These characteristics were reportedly beneficial to the material's toughness and strength.

## Nomenclature

CT: Cutting Temperature
SR: Surface Roughness
MV: Machine Vibration
SS: Spindle Speed
FR: Feed Rate
DC: Depth of Cut
ANOVA: Analysis of Variance
DOE: Design of Experiment
GRA: Grey Relational Analysis
GRC: Grey Relational Coefficient
GRG: Grey Relational Grade
MQL: Minimum Quantity Lubrication
SNR: Signal-to-Noise Ratio
HSS: High-Speed Steel
DOF: Degree of Freedom
MOS: Mean of Squares
SOS: Sum of Squares
M2: Tungsten-molybdenum high-speed steel

## 1 Introduction

During machining activities, an enormous amount of heat accumulates at the tool-work or tool-chip interface [[1], [2], [3]]. The distortion of the metal in the shear region in the path of the cutting edge, the physical separation of the metal at the point of separation, and the rubbing action of the chip, as it contacts over the tool's surface as it gets pushed out of the way, are the three processes that generate heat [[4], [5], [6], [7]]. The two main types of tool wear caused by heat generation are crater wear and flank wear, which limit the life of the tool and lead to dimensional inaccuracies, surface deterioration, and extreme corrosion situations on the workpiece [8]. The heat at the cutting region can be decreased by selecting the proper cutting settings, selecting the appropriate cutting instruments, and applying cutting fluids [9,10]. Cutting fluids are positioned between two moving parts to reduce wear, boost efficiency, and reduce surface friction. Cutting fluids can be made from chemical compounds, fixed oils, and mineral oils [11,12]. The most widely used cutting fluid is mineral oil [13,14]. Due to the number of negative environmental problems and consequences on operator health, mineral oil's numerous advantages have lately come under criticism [15]. Mineral oils are particularly hazardous and not sustainable because they contain intricate chemical substances that need to be treated before discharge. As a result, the procedure becomes more costly and complicated [16]. Furthermore, 7–17 % of the total manufacturing expenditure is spent on cutting fluids. Approximately 38 million tons of lubricants are used by manufacturers globally, with an expected increase of 1.2 % each decade [7]. The fluids are complicated chemicals; thus, disposal is a big problem. Researchers are investigating the MQL technique in this case to lessen the amount of cutting fluids needed during machining [17,18]. This technique can improve the cutting fluid's ability to travel through the cutting area while using less cutting fluid overall. Cutting fluids derived from vegetable oil, however, are often utilized because of their exceptional biodegradability, little negative effects on the ecosystem, and absence of contaminants. As a result, the combination of MQL and vegetable oil opens up a wide range of exciting possibilities.

Numerous studies have looked into the machining characteristics of cutting fluids made from vegetable oil. Agrawal and Patil [19] assessed the effectiveness of aloe vera oil as a lubricant in M2 steel machining with carbide cutting tool insert. Aloe vera oil outperformed mineral oil in terms of tool wear and surface finish. Nasution et al. [20] compared the efficacy of a commercially available soluble oil emulsion to the effects of raw palm oil and coconut oil on SR and tool wear. The findings revealed that the tool wear in the context of coconut oil was less than that of raw palm oil cutting fluid. Sharma and Sidhu [21] gazed into the effects of using a vegetable oil lubricant during dry and nearly dry machining of AISI D2 steel with a tungsten carbide insert. In terms of CT and SR, the results of the experiments demonstrated that near-dry machining performed better than dry machining. Junior et al. [22] investigated the technical potential of milling AISI 1045 steel with sustainable vegetable oils in an MQL operation. Among food-grade vegetable oils studied were canola, cottonseed, sunflower, babassu nut, soybean, and corn oils. Canola and cottonseed outperformed the others in tests to determine tool life during milling. Makhesana et al. [23] used castor oil and a coated carbide insert to turn AISI 4140 while running under MQL. The most effective outcomes are obtained by using a low FR, a high cutting speed, and a greater DC. Saleem and Mehmood [24] reported on the efficacy of an MQL technique using vegetable oils to accurately machine Inconel 718. The performances of castor and sunflower oils were evaluated and contrasted with the dry method. It was found that FR accounted for 97.86 %, 95.71 %, and 93.19 %, respectively, of SR in MQL castor oil, MQL sunflower oil, and dry machining environment. Burton et al. [25] utilized emulsified vegetable oil in water using ultrasonic atomization without surfactant. The potency of vegetable oil was tested directly in milling procedures. The performance of the vegetable oil surpassed the conventional oil in terms of cutting forces, chip thickness, and burr generation. Guntreddi and Ghosh [26] used an insignificant quantity of lubricant aerosol produced from air-atomizing sunflower seed oil at an intensity of 100 mL/h in a high-speed turning process. When juxtaposed to a dry setting, a tiny amount of oil could decrease cutting force by 5 %–20 %. Bork et al. [27] attempted the functionality of jatropha soluble oil in comparison with synthetic jatropha ester, canola oil, and the semisynthetic mineral during milling of the aluminum alloy 7050-T7451. Jatropha oil outperformed other oils in terms of lubrication requirements and shape errors. Shreeshail et al. [28] investigated the effects of groundnut oil and soybean oil during the milling of mild steel and aluminum alloy. In lubricating circumstances, more cutting force was required to mill a metal to achieve a better surface quality than it was in dry environments. Chinchanikar et al. [29] investigated the influence of dry, coconut oil-based, and water-based cutting fluids on hardened AISI 52100 steels during turning. Coconut oil proved to be superior in decreasing SR at higher DC and FR values. Moreover, Tazehkandi et al. [30] studied the effect of machining settings on SR, forces, and temperature during Inconel 706 tuning utilizing two ways of fluid applications, mineral oil in flooding mode and mist of sustainable vegetable oil with pressurized air. The results showed that SR, cutting forces, and cutting region temperature were substantially lower in all mist mode studies than in flooding mode trials. Elmunafi et al. [31] assessed the effectiveness of MQL during the processing of hardened stainless steel while using dry cutting and castor oil. It was discovered that adding a tiny amount of lubricant—50 mL/h—yields a greater tool lifespan than dry cutting. Pervaiz et al. [32] examined the effects of various lubricating flow conditions while utilizing TiAlN-PVD coated tools to machine Ti6Al4V. At a low FR of 0.1 mm/rev, it was found that raising the flow rate of oil from 70 mL/h to 100 mL/h enhanced the surface finish and decreased thermal softening. Makhesana et al. [33] examined the operation of MQSL and VMQL in the process of turning AISI 4140 steel using coated carbide tools. The results demonstrated the machining capability of VMQL and MQSL. The heat conductivity and viscosity of three vegetable oils were studied experimentally by Okafor and Nwoguh [34] using refined low oleic soybean oil, modified high oleic soybean oil, acculube LB2000 oil and mineral oil emulsion. The findings demonstrated that the viscosity of mineral oil was substantially lower than that of all vegetable oils, which all exhibit a sharp drop in viscosity with temperature. In terms of the direction of the fiber and milling FR, Elgnemi et al. [35] investigated the effectiveness of an oil-in-water emulsion produced by ultrasonic atomization without the use of a surfactant. Wet milling's efficiency is contrasted with a dry milling procedure. When compared to dry machining, using dispersed vegetable oil dramatically reduces cutting force, fiber delamination, and tool wear. Furthermore, Khunt et al. [36], demonstrated the efficacy of the assessment of vegetable based MQL in aluminum alloy drilling. These experiments were carried out under flood cooling, dry machining, MQL with sunflower oil, and MQL with castor oil. The juxtaposition of findings revealed that the vegetable oil was beneficial in terms of increased surface quality and reduced torque. Ajay Vardhaman et al. [37] investigated the influence of cutting fluids on friction coefficient, tool wear, surface finish, and chip structure during AISI 1040 steel turning. The MQL with coconut oil exhibited a considerable reduction in friction coefficient and tool wear, as well as an attractive chip structure and surface finish. When drilling AISI 304L stainless steel with a carbide tool, Puttaswamy et al. [38] studied the effects of Mahua (*Madhuca indica*), neem (*Azadirachta indica*), and conventional servo cut 945 mineral oil on thrust force, temperature, SR, and tool wear. Mahua and Neem oils offer a lot of potential as a substitute for traditional cutting fluids, according to findings. Pervaiz and Samad [39] used PVD-TiAlN coated carbide tools to assess the machining ability of Ti6Al4V alloy. They discovered that employing vegetable oil has the potential to minimize CT, SR, cutting forces, and harmful effects on the environment.

Based on assessments of the published works, the majority of the vegetable oils used are edible and could eventually compete with food-grade oil which serves human consumption. There has been little investigation into the use of non-edible vegetable oils as cutting fluids. Therefore, this research examines the influence of MQL watermelon oil on CT, SR, and machine vibration rate during turning AISI 1525 steel at different feeds, SSs, and depths of cut. Taguchi GRA was used to identify optimum process parameters. Furthermore, the process proceeds with the response surface generation, contour plots as well as a mathematical model based on the data generated from the experiment.

## 2 Materials and methods

### 2.1 Watermelon oil extraction and characterization

The watermelon seeds used in this study were obtained from a central market in Argungu, Kebbi state, Nigeria. The sun naturally dried the watermelon seeds for six days at an approximate temperature of 35 °C on average before it was eventually crushed. The particle size of crushed seeds is typically between 0.2 mm and 0.5 mm. N-hexane was used for the extraction of oil, which took place at 60 °C for 140 min. Testing for phytochemical, physiochemical, and lubricity-related oil qualities was carried out on crude oil extract. The tests were carried out to assess each of the oils' compositional features and to identify the chemical groups present in the sample. All of these are essential to establish the effectiveness of the oil for a certain system. The procedures and results for the different variables determined on the raw watermelon oil had been published by Kazeem et al., [40].

### 2.2 Selection of cutting tool and workpiece

**Fig. 1:**
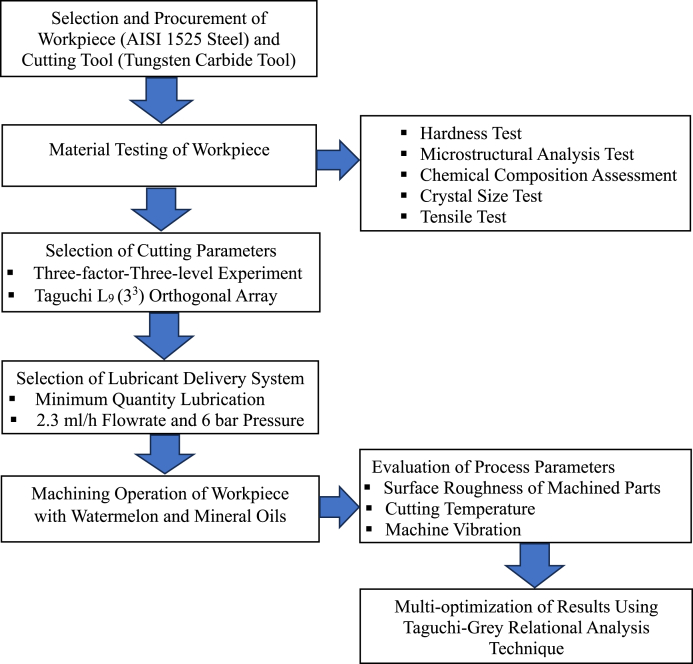
Flowchart for machining process and optimization.

For the turning procedures in the present investigation, solid cylindrical bars (measures 40 mm in diameter and 120 mm in length) made of medium carbon steel, AISI 1525, were chosen. AISI 1525 steel was considered due to its adaptive qualities, such as outstanding endurance against chloride corrosion, water resistance, and high strength at extreme temperatures. AISI 1525 steel is used in critical sectors such as nuclear power stations, pressure vessels, boilers, chemical manufacturing services, gas turbines and furnaces, and maritime technology. A tungsten carbide insert tool (insert model: CNMG12040408) was used as the cutting insert. The machining operation was conducted on a typical AJAX lathe. The system adopts an MQL delivery technique with an air pressure was 6 bar and an oil flow rate of 2.3 mL/h [41]. Three input factors, namely SS, DC, and FR were considered for the experiments. The study was conducted using the Taguchi DOE. Three levels were considered for each of the aforesaid input parameters, as given in Table 1. Therefore, Taguchi specified L_9_ (3^3^) orthogonal configuration for a three-factor, three-level experiment. The flowchart that best describes the methodology is displayed in Fig. 1. The photographic view of the experimental process is shown in Fig. 3 *(a-f).*

**Table 1:** Factor and levels of the orthogonal array.

Variable	Level		
	Level 1	Level 2	Level 3
FR(mm/rev)	0.10	0.15	0.20
SS(rev/min)	355	500	710
DC(mm)	0.75	1.00	1.25

### 2.3 Steel chemical composition determination using optical emission spectrometer (OES)

An OES was used to assess the chemical constituents of AISI 1525 steel. For situations where analyzing the precise volumetric breakdown of a solid substance is necessary, the OES is the best option. This method calculates the elemental abundance based on spectral sensitivities using the optical emission spectra typical of a given element [42,43]. It has the benefit of allowing for the simultaneous determination of the abundance of several elements of interest in the sample, such as all first-row transition metals. The OES requires applying a current to the specimen while also vaporizing an insignificant quantity of material. When a spark happened, a discharge plasma with a specific chemical signature emerged, which could be used to determine the specimen's elemental composition [44].

### 2.4 Steel mechanical properties determination

A digital Instron electromechanical global tensile testing machine was used to perform the tensile test on the specimen. This testing apparatus includes a computer, test software, and printer. This apparatus helps to provide an accurate assessment of the mechanical characteristics of the metal material, including its tensile and yield strength. A piece of AISI 1525 steel was first turned into an appropriate gauge measurement on a central lathe in preparation for the tensile test [45]. The specimens were attached to the load-carrying cell chamber of the device, which contained transducers that converted the applied load into electric impulses and communicated the results to the computer. The measurement was conducted by moving the specimen in various positions till it broke.

### 2.5 Steel hardness test determination

Indentation testing and scratch testing are the two main techniques for determining the hardness of a material [46]. Only components that can bend plastically, like thermoplastic polymers and metals, can be tested by indentation. To evaluate fragile materials like ceramics, scratching is employed. In this study, indentation testing was considered to determine the hardness of the workpiece. Before subjecting the specimen to hardness tests, it was ground and polished with a polish machine to achieve a smooth and shiny surface [47]. The machine was equipped with a compression connection, the compression dies, and indenter were inserted, and the mercury gauge was reset to zero. After positioning the flat surface onto the indenter, the load was applied until the mercury gauge registered 200 kg. The load was released from the specimen after a few moments by removing the compression connection. The circumference of the ball's impression was acquired with a calibrated hand lens, and the associated Brinell hardness number was obtained. The hardness of the workpiece was carried out at every 5 mm increase of the workpiece. The Brinell hardness number is expressed using Eq. (1).

(1) $$BHN=\frac{2F}{\pi d_{i}\left[D_{b}-\sqrt{{D_{b}}^{2}-{d_{i}}^{2}}\right]}$$

where F= Load applied (kgf), $D_{b}=$ Ball diameter (mm), $d_{i}=$ Indentation diameter (mm).

### 2.6 Microstructural analysis of AISI 1525 steel

The microstructure of the workpiece was examined to ascertain whether there were microstructural deformations or microstructural changes in the workpiece before machining [48]. The microstructure was carried out at 70 mm, 35 mm, and 5 mm diameters of the workpiece. The microstructure was carried out with a metallurgical microscope. The magnification employed was 200×.

Selection and Procurement of Workpiece (AISI 1525 Steel) and Cutting Tool (Tungsten Carbide Tool).

### 2.7 Determination of number of grains in AISI 1525 steel

This was carried out to be sure of the consistency in the mechanical properties of the workpiece material. This was evaluated at three different points of the workpiece. The three points are at 70 mm, 35 mm, and 5 mm diameter of the workpiece. The ASTM expression relating the number of grains per inch square at 100× is given by Eq. (2). A test circle with a diameter of 79.8 mm was positioned over the microscopic structure in the planimetric grain size method. To count at least 100 grains, a magnification is used. The calculation is given by Eq. (3).

(2) $$N_{A}=2^{n-1}$$

where $N_{A}=$ number of grains per inch square at 100X and n=ASTM grain size number.

(3) $$N_{A}=f\left(\eta_{inside}+0.5\eta_{\mathrm{int}ercepted}\right)$$

where *N*_*A*_ = number of grains per mm^2^ at *1X*, *f* = planimetric multiplier, *n*_*inside*_ number of grains that are completely inside the test circle*, n*_*intercepted*_ = number of grains that are intercepted by the circle.

### 2.8 Equipment for measurements

**Fig. 2:**
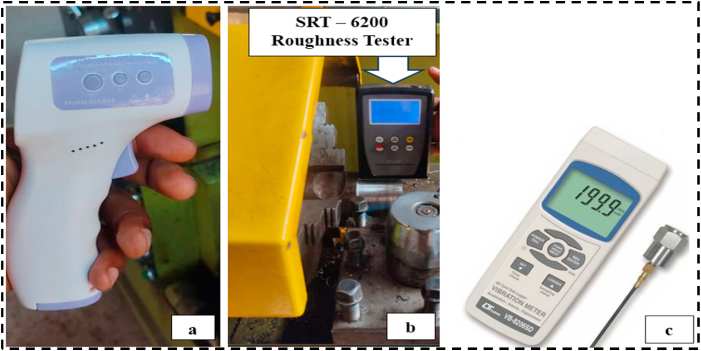
Measuring Equipment Utilized (a) PeakTech Infrared thermometer (b) SRT – 6200 roughness tester (c) Lutron Vibration Meter VB8206SD.

Turning temperature is a characteristic that has a major impact on the surface characteristics and life span of the workpiece; lower temperatures suggest better efficiency. The CT was examined with a PeakTech Infrared thermometer (shown in Fig. 2(a)) with an emissivity of 0.95. The instrument has a 2 % accuracy and measures temperatures ranging from −50 to 380 °C. During machining, the thermometer's tip is pointed to the chip-tool interface to measure the amount of heat there. The tool-chip interface and the infrared temperature sensor were manually held around 5 cm apart. Three readings were taken for each sample, from which the average reading was calculated. However, the workpiece's SR is a significant metric for assessing turning machining qualities, and a lower value of SR indicates good workpiece serviceability [49]. The SR of the workpiece was assessed using a Mextech SRT-6200 detector shown in Fig. 2(b). The measurement was taken at three random spots after every test, and an average level was calculated. The device has a 10 % precision and a working temperature range of 0–50 °C. In this equipment, a detector is positioned on the outermost layer and then consistently moves along the surface by driving the device within the tester. The sharp built-in probe of the roughness tester sensor analyzes SR. Vibrations, on the other hand, are unavoidable throughout the machining process and have numerous undesirable repercussions, the most significant of which are as follows: Machining dependability is compromised by excessive wear on cutting edges as well as unpredictable, uncontrollable wear patterns (such as chipped and cracked cutting edges). The Lutron vibration meter (VB8206SD) used to measure the machine tool's vibration in various cutting conditions is depicted in Fig. 2(c). The device has an accuracy of ( ± 5 %). The vibration meter probe was positioned adjacent to the spindle on the machine's headstock. The meter measures displacement, velocity, and acceleration, but for this study, the reading was taken in terms of acceleration (m/s^2^). It is capable of continually recording the minimum and maximum spindle vibration (sensor data) [40].

**Fig. 3:**
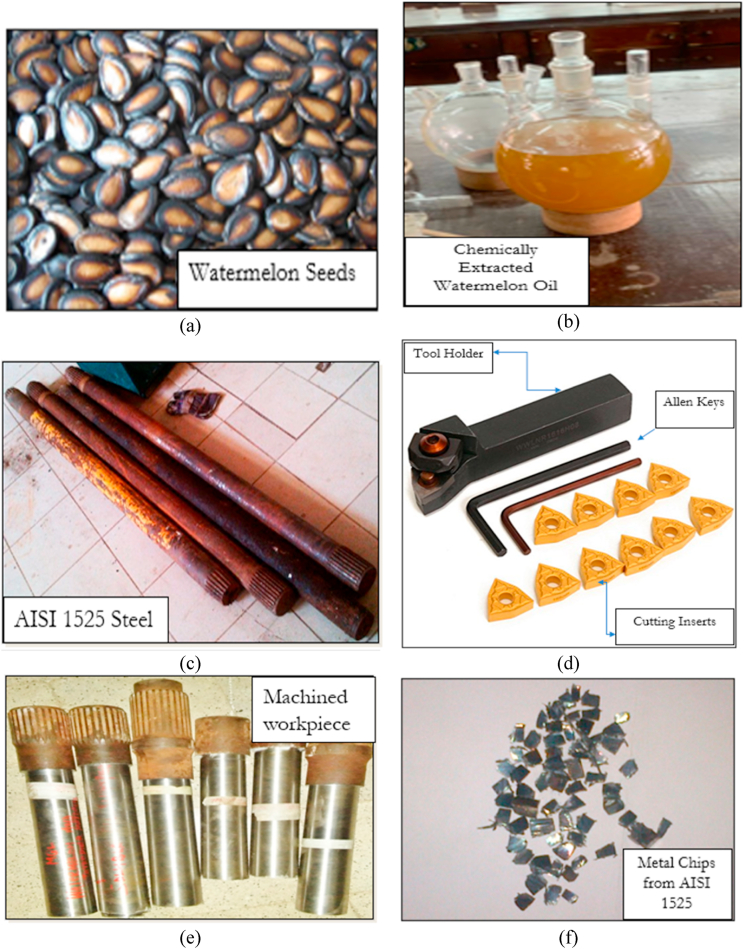
Photographic view of experimental process (a) Watermelon seeds used for the study (b) extracted watermelon crude oil (c) Scrap AISI 1525 steel (d) Cutting tools and tool holder (e) Machined AISI 1525 steels (f) Metal chips obtained from machining operation.

### 2.9 Signal to noise ratio analysis

The SNR values for both CT and SR were determined and optimized. According to Eq. (4), the superior characteristic used in this study for CT and SR was a lower SNR.

(4) $$SNR=-10\;\log\frac{1}{n}\left(\sum {y_{i}}^{2}\right)$$

Where SNR = signal-to-noise ratio; y = the measure quality characteristic for the $i^{th}$ repetition, and n = number of repetitions in a trial.

### 2.10 Multiple regression model analysis

Mathematical models were generated to predict process parameters by using multiple regression analysis which was carried out on Minitab software.

### 2.11 Assessment of surface and contour plots

The relationship between the SR, CT and machine vibration was examined and interpreted through contour plots and response surface methodology.

## 3 Results and discussion

### 3.1 Material testing evaluation

**Fig. 4:**
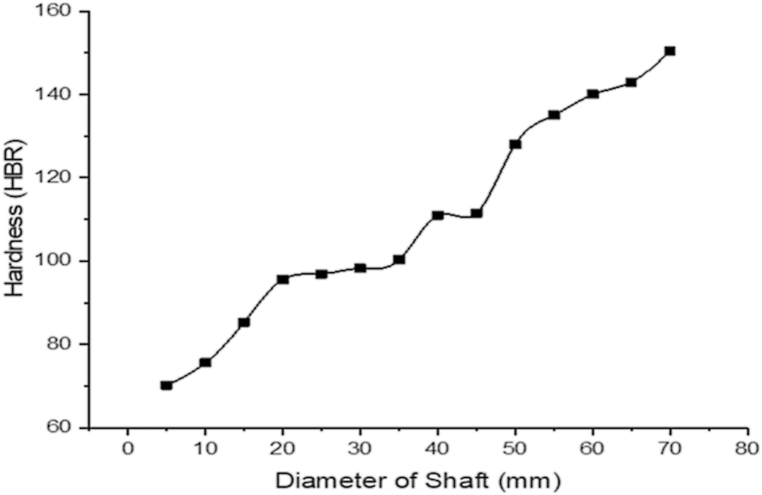
Hardness of AISI 1525 steel material.

The chemical constituent in the workpiece includes C (0.2505), Si (0.2215), Mn (1.2340), S (0.0235), P (0.0135), Cr (0.1135), Ni (0.1155), Cu (0.0480), Nb (0.0001), Al (0.0120), Ti (0.0395), Fe (97.9285) and Remaining (0.0003). Iron has the largest composition of all. The steel is designated as AISI 1525 steel based on AISI/SAE requirements and the specified elements. The yield strength, tensile strength, elastic modulus, energy at break, and energy at maximum tensile stress were measured as 7599.34 MPa, 604.74 MPa, 35106.19 MPa, 47.35634 J, and 31.24909 J, respectively. The hardness of AISI 1525 steel was carried out at every 5 mm diameter of the steel and the result is represented in Fig. 4. The hardness increases with an increase in diameter. This implies that the workpiece material is less hard at every 5 mm reduction of the workpiece diameter due to the decline in the indentation resistance of the workpiece [50,51]. Generally, the cross-sectional area of a steel rod is directly related to its hardness and tensile strength. As the diameter of the steel rod increases, the cross-sectional area of the rod also increases [52,53]. Hence, the hardness, which is roughly proportional to tensile strength also increases. Moreover, the diameter of a steel rod could affect stress distribution on it [54,55]. Therefore, with a larger diameter, there is a likelihood of a more uniform stress distribution, which could result in a reduction in the localized stress concentration and consequently enhance the hardness and strength of the steel rod [56,57]. Also, the image analysis performed across the steel validated that the workpiece is less hard as it descends across its diameter.

**Fig. 5:**
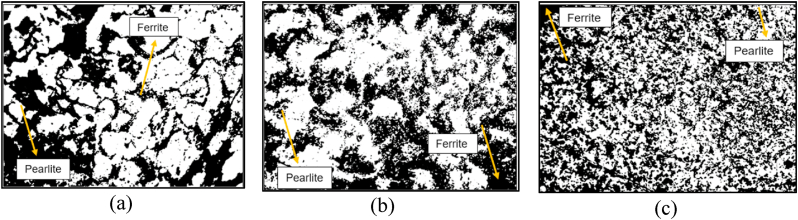
Microstructure of steel at (a) 5 mm shaft diameter (b) 35 mm shaft diameter and (c) 70 mm shaft diameter.

The microstructure of the workpiece at 5 mm, 35 mm, and 70 mm of the shaft is shown in Fig. 5(a–c). The quantitative metallography of steel analyzed is shown in Table 2. The microstructure of the steel at 5 mm diameter shows both white and black areas. The white area represents the ferrite phase while the black area represents the pearlite phase. It has a distinct grain boundary and has a more ferrite phase than the pearlite phase. The microstructure of the steel at 35 mm diameter shows that there are equal amounts of pearlite and ferrite. At 70 mm in diameter, there are tiny and cluster phases of ferrite and pearlite. The grains are closer, and this particular steel has a higher hardness value because of the grain boundaries that are relatively close compared with 35 mm and 5 mm diameters. The finer grain sizes at 70 mm diameter could be beneficial to the material's toughness and strength [58,59]. According to the assertion of Ke et al. [60], and Shams et al. [61], finer grain sizes provide superior grain boundaries, improve deformation resistance, and impede dislocation movement. These are some of the factors which affect the mechanical performance of steel. The predominant presence of pearlites at 70 mm diameter relative to the 5 mm and 35 mm diameters could also be the reason for the high hardness it exhibited, since pearlites are hard and strong, while ferrite is soft [62,63]. The quantity of grains per square inch at 1× magnification can be calculated using the grain size specifications provided by the ASTM worldwide. The average grain size decreases as the grain-size number rises. Steels frequently have grain sizes between 10 and 12. Grain sizes for conventional low strength forming steels hover around 6 or 7. Grain sizes 5 and below can have visible surface issues including tears, cracks, and orange peels [64].

**Table 2:** Quantitative metallography of steel.

Steel Metallograph at	No. of Grain Inside	No. of Grain Outside	Grain Size Number	Type of Grain obtained
Ø5 mm	24	17	6.02	Coarse
Ø35 mm	26	18	6.13	Medium
Ø70 mm	90	60	7.91	Fine

**Fig. 6:**
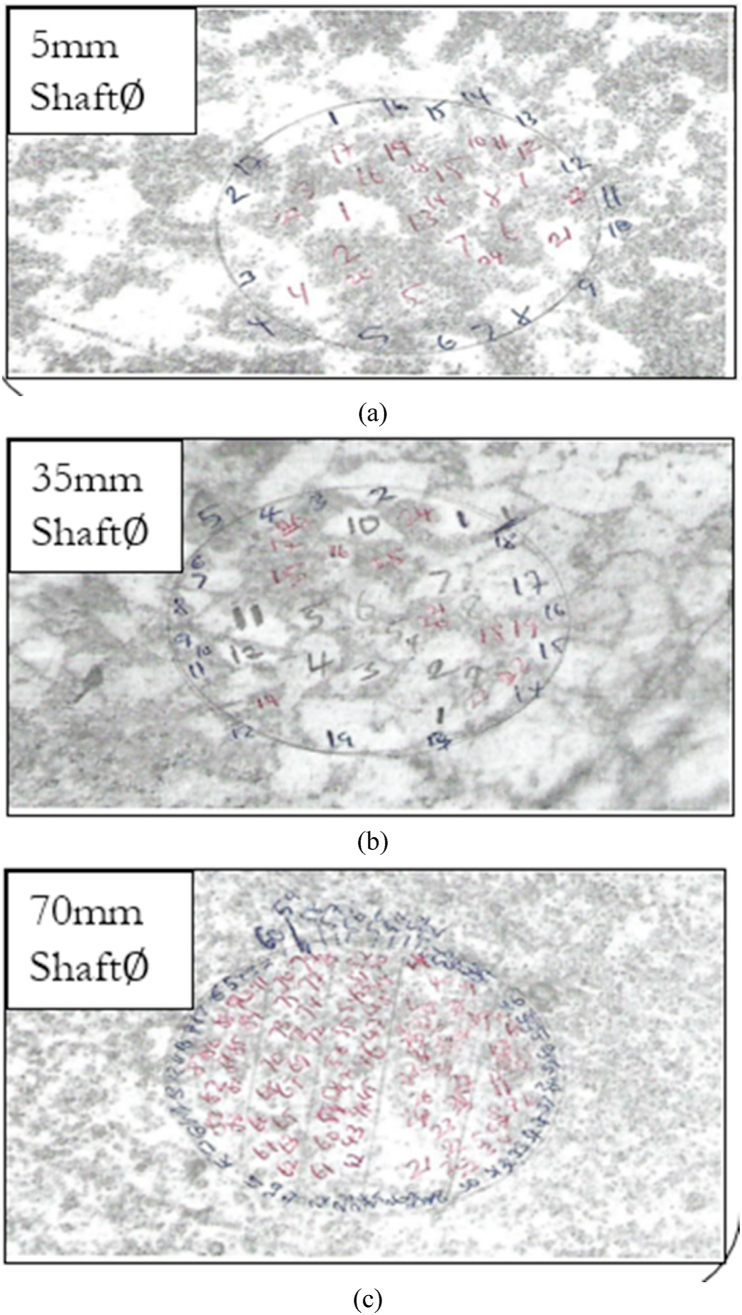
(a) Quantitative metallography of steel at 5 mm (b) Quantitative metallography of steel at 35 mm (c) Quantitative metallography of steel at 70 mm.

The boundaries of the grains resist deformation and enable the grain core to deform when the steel is stretched to high strain levels. This is not acceptable in the machining of materials. It is advisable to specify a grain size number of 6 or higher. Quantitative metallography of steel at 5 mm, 35 mm, and 70 mm is shown in Fig. 6 *(a-c)* and according to Table 2, the grain size number for the three steel metallographs is within the acceptable range (6.02–7.91). Grains in metals can be categorized into coarse-grained, medium-grained, and finer-grained materials. The 70 mm diameter shaft is a representation of a fine grain. A fine-grained structure has higher toughness [65]. A material's toughness is determined by its capacity to absorb energy without breaking. A decrease in grain size alters the mechanical characteristics of materials. Coarse-grained materials are less hard, have a lower yield strength, and are more ductile than fine-grained materials. Less cracking and tearing allows for more ductile material formation [66]. Large grains in excess might also be problematic. Greater resistance to dislocation is associated with more grain boundaries, which are created by finer grain sizes, as in the case of the 70 mm diameter shaft. It refers to a material's measurable capacity to endure significant plastic deformation, which renders the material less ductile.

### 3.2 Evaluation of machining environments

This section provides a summary of the findings from trials conducted using identical MQL parameters under the circumstances of mineral oil and watermelon lubrication. The impacts of the MQL variables (SS, FR, and DC) on the turning temperature, machine vibration, and SR were first assessed using the Taguchi-method analysis. The best MQL variable arrangement was then discovered using multi-objective optimization based on the GRA approach.

#### 3.2.1 Assessment of turning characteristics using the Taguchi method

**Fig. 7:**
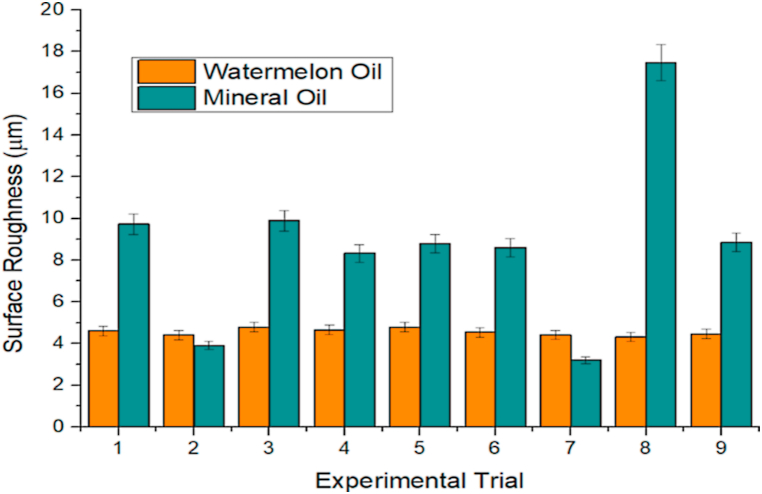
Effect of machining lubricants on SR

**Fig. 8:**
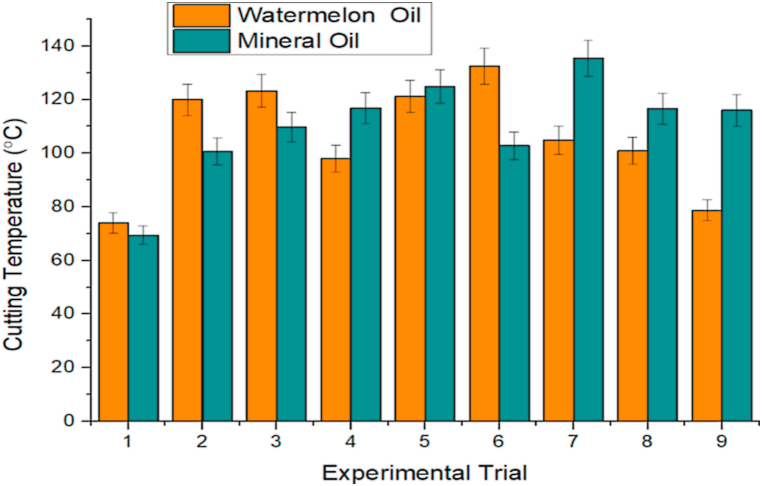
Effect of machining lubricants on CT

**Fig. 9:**
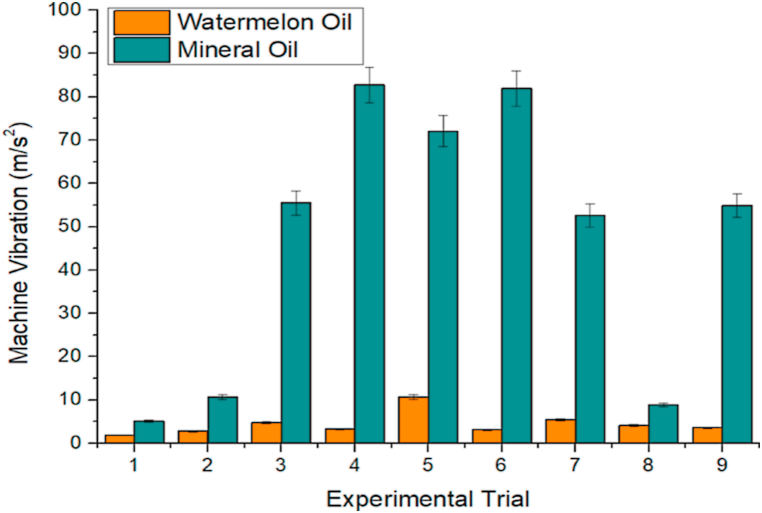
Effect of machining lubricants on machine vibration.

As earlier mentioned, the Taguchi method was used to conduct orthogonal experiments, and findings were gathered. Table 3, Table 4 show the orthogonal experiment setups and findings for watermelon and mineral oils, respectively. The effect of machining lubricants (watermelon oil and mineral oil) on SR is indicated in Fig. 7. In most of the experimental trials, lower SR was observed with the application of watermelon oil. For instance, in experiment 1, the SR with the application of mineral oil and watermelon oil was 9.72 and 4.6 μm, respectively. The aforementioned SR values indicated that approximately 52.67 % reduction in SR was achieved with the application of watermelon oil, relative to mineral oil. Similarly, in experiments 3, 4, 5, 6, 8, and 9, reductions in SR with the application of watermelon oil relative to the mineral oil were 51.57 %, 43.99 %, 45.57 %, 47.33 %, 75.33 %, and 49.60 %, respectively. These percentage reductions in SR showed that the watermelon oil possibly exhibited higher viscosity [67]. The low SR observed on the application of watermelon oil could be beneficial to the better surface finish and surface integrity of the workpiece [24,68]. In experiments 2 and 7, the SR was marginally lower with the application of mineral oil, relative to the watermelon oil, which could be a result of machine tool conditions and workpiece material properties [69,70]. The high SR that is largely observed in the application of mineral oil could affect surface-wetting characteristics and the dynamic angle of contact during the droplet's interaction with surfaces [71,72]. Fig. 8 further shows the effect of machining lubricants (watermelon oil and mineral oil) on CT. In experiments 1, 2, 3, and 6, the application of mineral oil resulted in a better cooling effect on the workpiece due to sufficient penetration of oil at the cutting interface. However, in the other experiments (4, 5, 7, 8, and 9), watermelon oil significantly affected the CT. This indicated that the watermelon oil minimized heat generation and consequently reduced the friction between the workpiece and the cutting tool [73,74]. The reduction in friction is advantageous to surface quality, enhances machining efficiency, and prolongs tool life [75,76]. The heat generation, if not reduced could also cause flank wear and crater, leading to a decrease in the lifespan of a cutting tool, surface deterioration, dimensional inaccuracy, and severe cases of corrosion effect on the workpiece [77,78]. The effect of machining lubricants (watermelon oil and mineral oil) on machine vibration is shown in Fig. 9. It is worthy of note that the watermelon oil drastically reduced the machine vibration, compared to the mineral oil. The effect is most noticeable in experiments 4 and 5, where the percentage decrease in the machine vibration on the application of watermelon oil was 96.11 m/s^2^ and 96.23 m/s^2^, respectively, compared to the mineral oil. The machine vibration reduction performance of the watermelon oil could be attributed to its ability to absorb heat energy and provide some degrees of damping on the cutting tools and the workpiece, which in turn reduced the effect of machine imbalance and also enhanced damping [[79], [80], [81]]. The watermelon seed oil contains a predominantly high percentage of unsaturated fatty such as Linoleic and Oleic which are responsible for their superior viscosity, molecular weight, and viscosity index [82]. Hence, enhancing the interaction between the workpiece and the cutting tool through the formation of an effective lubricant layer, which also leads to the absorption of heat and reduction of friction between the tool and the workpiece. In general, SR, CT, and machine vibration were 40.95 ± 0.172 μm, 953.4 ± 20.268 °C and 39.57 ± 2.585 m/s²; and 78.75 ± 4.075 μm, 992.2 ± 18.645 °C and 424.24 ± 31.255 m/s² for watermelon oil and mineral oil, respectively. Watermelon oil surpassed mineral oil in terms of machine vibration, SR, and CT. This study therefore considers the further evaluation of watermelon oil in the analysis of the main effects plot, ANOVA, signal-to-noise ratio, GRA, surface plot, and contour plot.

**Table 3:** Orthogonal experiment arrangements and results for watermelon oil.

MQL Input Parameters			Turning Characteristics		
DC(mm)	FR(mm/rev)	SS(rev/min)	CT(°C)	SR(μm)	$MV\left(m/s^{2}\right)$
0.75	0.10	355	74.0	4.60	1.82
1.00	0.15	355	119.9	4.40	2.79
1.25	0.20	355	123.3	4.79	4.75
1.00	0.10	500	98.0	4.66	3.22
1.25	0.15	500	121.2	4.79	10.66
0.75	0.20	500	132.5	4.53	3.09
1.25	0.10	710	104.9	4.41	5.45
0.75	0.15	710	100.9	4.31	4.18
1.00	0.20	710	78.7	4.46	3.61

**Table 4:** Orthogonal experiment arrangements and results for mineral oil.

MQL Input Parameters			Turning Characteristics		
DC(mm)	FR(mm/rev)	SS(rev/min)	CT(°C)	SR(μm)	$MV\left(m/s^{2}\right)$
0.75	0.10	355	69.4	9.72	5.12
1.00	0.15	355	100.6	3.90	10.6
1.25	0.20	355	109.7	9.89	55.5
1.00	0.10	500	116.8	8.32	82.7
1.25	0.15	500	124.9	8.80	72.02
0.75	0.20	500	102.8	8.60	81.9
1.25	0.10	710	135.4	3.20	52.6
0.75	0.15	710	116.6	17.47	8.9
1.00	0.20	710	116.0	8.85	54.9

**Fig. 10:**
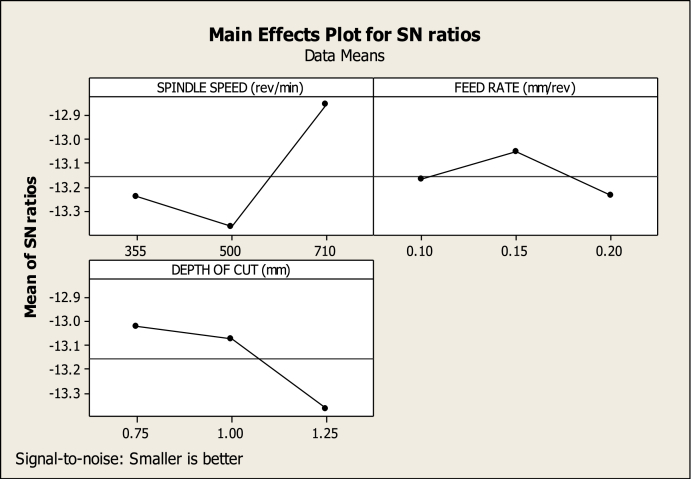
Factors effect on SR

**Fig. 11:**
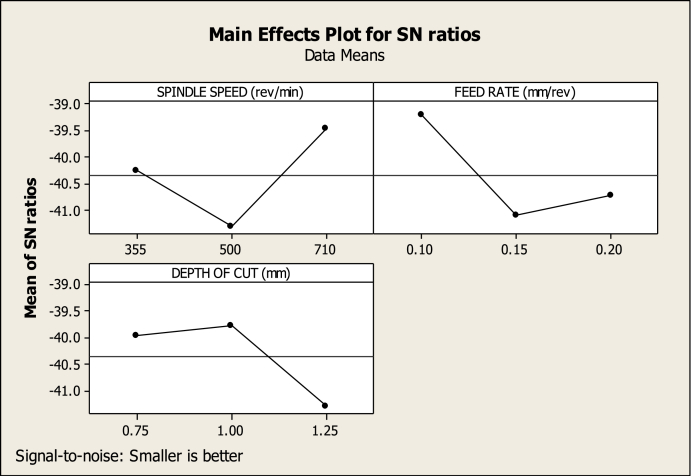
Factors effect on CT

**Fig. 12:**
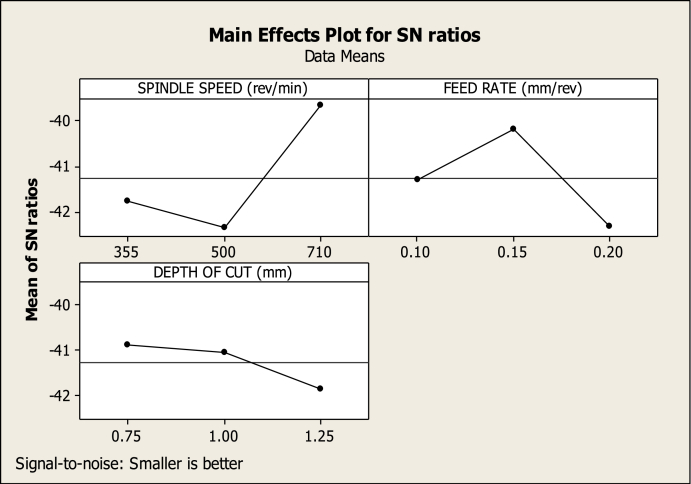
Factors effect on MV

Table 5 shows the ANOVA of the turning variables. The main effects plot for SR (Fig. 10) shows that 0.15 mm/rev FR (level 2), 710 rev/min SS (level 3), and 0.75 mm DC (level 1) have a greater impact on SR reduction than other SS, feed, and cutting depth combinations. Table 5 shows the part played by all the variables in lowering SR; it is clear that SS made the most significant impact, at 61.55462 %, which was followed by DC, at 24.73529 %. The other variables, on the other hand, make smaller contributions, making them less important. In the meantime, the ideal cutting settings for the CT (as shown in Fig. 11) are an SS of 710 revs per minute (level 3), an FR of 0.10 mm per revolution (level 1), and a DC of 1.00 mm (level 2). Also, in Table 5, the SS, FR, and DC all contribute differently to CT, with respective contributions of 22.9075 %, 53.09988 %, and 15.7839 %. Therefore, throughout the turning process, SS and FR have a substantial impact on the CT. The effect of cut depth is less substantial. Fig. 12 shows the optimum parameters for MV as 710 rev/min, 0.15 mm/rev, and 0.75 mm. The amount of contribution from each process variable is also shown in Table 5. The chosen confidence level for the ANOVA is 95 %.

**Table 5:** ANOVA for SR, CT, and MV.

Factor	Variable	DOF	SOS	MS	F	P%
SR(μm)	FR(mm/rev)	2	0.01327	0.006635	0.685434	5.57563
	SS(rev/min)	2	0.1465	0.07325	7.567149	61.55462
	DC(mm)	2	0.05887	0.029435	3.040806	24.73529
	Error	2	0.01936	0.00968		8.134454
	Total	8	0.238	0.02975		100
CT(°C)	FR(mm/rev)	2	1745	872.5	6.468713	53.09988
	SS(rev/min)	2	752.8	376.4	2.790629	22.9075
	DC(mm)	2	518.7	259.35	1.92282	15.7839
	Error	2	269.76	134.88		8.208724
	Total	8	3286.26	410.7825		100
$MV\left(m/s^{2}\right)$	FR(mm/rev)	2	25.48	12.74	8.039833	51.64981
	SS(rev/min)	2	16.15	8.075	5.095891	32.73723
	DC(mm)	2	4.533	2.2665	1.43032	9.188721
	Error	2	3.16922	1.58461		6.42424
	Total	8	49.33222	6.166528		100

#### 3.2.2 Evaluation of turning characteristics based on the GRA

The grey number theory, which includes GRA, is appropriate for resolving issues involving complex relationships between variables and numerous factors [83]. In some attribute decision-making issues, the GRA has been effectively applied. The most desirable characteristics can be obtained by using GRA to determine the optimal state of various input parameters. The GRA is frequently used to assess or judge the effectiveness of a complex project with scant data. By assigning weights to each of the responses, GRA can be used to determine the ideal conditions for multi-objective problems. Below is a list of the steps in a GRA.

1 Data Pre-processing and Normalization

The data for grey analysis must be preprocessed into quantitative indices to normalize raw data for further analysis. Data preprocessing is the process of transforming a raw data sequence into a decimal sequence ranging from 0.00 to 1.00 for evaluation. If the anticipated data sequence is of the form “Higher-the-better”, the original sequence can be normalized using Eq. (5).

(5) $${s_{i}}^{*}\left(k\right)=\frac{{s_{i}}^{ο}\left(j\right)-\min\;{s_{i}}^{ο}\left(j\right)}{\max\;{s_{i}}^{ο}\left(j\right)-\min\;{s_{i}}^{ο}\left(j\right)}$$

Where ${s_{i}}^{ο}\left(j\right)$ is the original sequence, ${s_{i}}^{*}\left(j\right)$ the sequence after the data preprocessing, $\max\;{s_{i}}^{ο}\left(j\right)$ the largest value of ${s_{i}}^{ο}\left(j\right)$, and minimum $\min\;{s_{i}}^{ο}\left(j\right)$ implies the smallest value of ${s_{i}}^{ο}\left(j\right)$. When the form “Smaller-the-better” becomes the expected value of the data sequence, the original sequence can be normalized using Eq. (6).

(6) $${s_{i}}^{*}\left(k\right)=\frac{\max\;{s_{i}}^{ο}\left(j\right)-\;{s_{i}}^{ο}\left(j\right)}{\max\;{s_{i}}^{ο}\left(j\right)-\min\;{s_{i}}^{ο}\left(j\right)}$$

In machining processes, the desired response was a minimum SR, MV, and CT, so a ‘smaller the better’ normalization equation was chosen. Table 6 shows the individual responses to normalization.

2 Deviation Sequence

**Table 6:** Pre-processing of data.

Exp.	SNR			Normalizing Sequence		
	SR(μm)	CT(°C)	$MV\left(m/s^{2}\right)$	SR(μm)	CT(°C)	$MV\left(m/s^{2}\right)$
1	−13.255	−37.385	−5.201	0.617	0.000	0.000
2	−12.869	−41.576	−8.912	0.196	0.828	0.242
3	−13.607	−41.819	−13.534	1.000	0.876	0.543
4	−13.368	−39.825	−10.157	0.739	0.482	0.323
5	−13.607	−41.670	−20.555	1.000	0.847	1.000
6	−13.122	−42.444	−9.799	0.471	1.000	0.299
7	−12.889	−40.416	−14.728	0.217	0.599	0.620
8	−12.690	−40.078	−12.424	0.000	0.532	0.470
9	−12.987	−37.919	−11.150	0.324	0.106	0.387

The deviation sequence of the reference sequence is expressed using Eq. (7).

$$\nabla_{oi}\left(j\right)=\left\|{s_{o}}^{*}\left(j\right)-{s_{i}}^{*}\left(j\right)\right\|$$

$$\nabla_{\max}=\max_{\Psi k\varepsilon i}\max_{\Psi j}\left\|{s_{o}}^{*}\left(j\right)-{s_{k}}^{*}\left(j\right)\right\|$$

(7) $$\nabla_{\min}=\min_{\Psi k\varepsilon i}\min_{\Psi j}\left\|{s_{o}}^{*}\left(j\right)-{s_{k}}^{*}\left(j\right)\right\|$$

ζ is the distinguishing coefficient. ζε[0,1]. ζ = 0.5 is generally used.

3. GRC

To express the relationship between the ideal and actual normalized experimental results, the GRC is calculated. Eq. (8) is used to express the GRC as a result.

(8) $$\zeta_{i}\left(j\right)=\frac{\nabla_{\min}+\zeta \cdot \nabla_{\max}}{\nabla_{oi}\left(j\right)+\zeta \cdot \nabla_{\max}}$$

Where $\nabla_{oi}\left(j\right)$ is the deviation sequence of the reference sequence.

4. GRG

The average of the GRC is typically taken as the GRG after the GRC has been determined. Using Eq. (9), the GRG is described.

(9) $$\gamma_{i}=\frac{1}{n}\sum_{j=1}^{n}\zeta_{i}\left(j\right)$$

The deviation sequence, GRC, and GRG values are shown in Table 7 following careful value entry into Eq (7), (8), (9). According to the values of the GRG, the rank, *rk(A),* was assigned.

**Table 7:** Grey relational analysis.

Exp.	Deviation Sequence			GRC			GRG	rk(A)
	SR(μm)	CT(°C)	$MV\left(m/s^{2}\right)$	SR(μm)	CT(°C)	$MV\left(m/s^{2}\right)$		
1	0.383	1.000	1.000	0.566	0.333	0.333	0.305	9
2	0.804	0.172	0.758	0.383	0.745	0.397	0.381	5
3	0.000	0.124	0.457	1.000	0.802	0.522	0.581	2
4	0.261	0.518	0.677	0.657	0.491	0.425	0.393	4
5	0.000	0.153	0.000	1.000	0.766	1.000	0.691	1
6	0.529	0.000	0.701	0.486	1.000	0.416	0.476	3
7	0.783	0.401	0.380	0.390	0.555	0.568	0.378	6
8	1.000	0.468	0.530	0.333	0.517	0.486	0.334	7
9	0.676	0.894	0.613	0.425	0.359	0.449	0.308	8

**Fig. 13:**
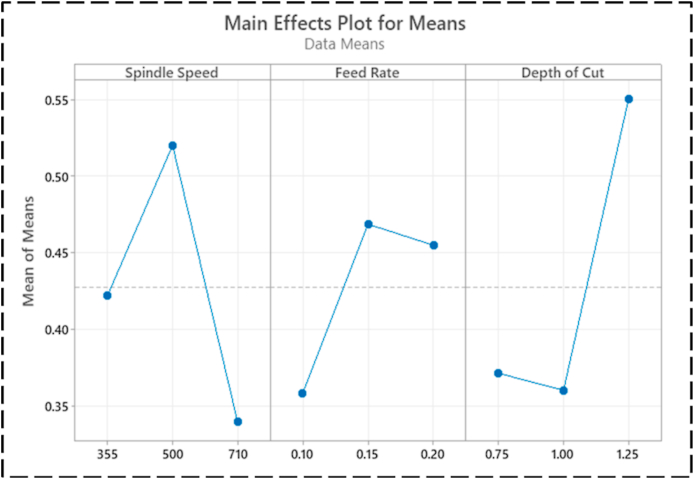
Main effects plot for GRG for the process parameter.

Table 7 shows that experiment 5 has the highest value of GRG and was assigned rank one. The variables in the setup for experiment number 5 have an FR of 0.15 mm/rev, an SS of 500 rev/min, and a DC of 1.25 mm. The grey grade values in Table 7 vary according to the experiment number. An analysis utilizing ANOVA of the GRG data was conducted to determine the statistical significance of each of the three input parameters. To find out how the experimental variables affected the outcomes of the different performance components, an ANOVA test was run, as indicated in Table 8. SS, FR, and DC had a 35 %, 15.5 %, and 48.69 % influence on GRG levels, respectively, according to the results of the ANOVA in Table 8. Thus, the most significant factor affecting GRG levels was the DC. Table 9 shows the grey relationship between the mean GRG values for the process parameter and the level. Selecting the optimal process parameter considers the three response factors (FR, SS, and DC). The ranking shows the relative importance of each parameter on the result. FR has minimal impact on the result, but DC has the biggest. The best parameters can be visually identified using the main effects plot for GRG. When turning AISI 1525 steel with a tungsten carbide tool, as illustrated in Fig. 13, the FR (level 1), SS (level 3) and DC (level 2) all have a considerable favorable effect.

**Table 8:** Results of ANOVA on GRG for watermelon oil lubricant.

Source	DOF	SeqSOS	Contribution	AdjSOS	AdjMOS	F−value	P−value
FR(mm/rev)	2	0.021567	15.50 %	0.021567	0.010783	19.21	0.049
SS(rev/min)	2	0.048718	35.00 %	0.048718	0.024359	43.39	0.023
DC(mm)	2	0.067771	48.69 %	0.067771	0.033885	60.35	0.016
Error	2	0.001123	0.81 %	0.001123	0.000561		
Total	8	0.139178	100.00 %

**Table 9:** The GRG response table for watermelon oil.

Parameter	Level−1	Level−2	Level−3	Delta	rk(A)
FR(mm/rev)	0.3587	0.4687	0.4550	0.1100	3
SS(rev/min)	0.4223	0.5200	0.3400	0.1800	2
DC(mm)	0.3717	0.3607	0.5500	0.1893	1

### 3.3 Regression analysis

Regression models are statistical instruments employed to examine the relationship between a dependent factor and several independent variables [84]. Using the variables FR, SS and DC, a multiple regression model with a 95 percent confidence level for both responses—that is, SR, MV, and CT—was constructed. The value of the model's coefficients of determination was calculated in order to evaluate the suitability of the model (R^2^); R-squared values normally range from 0 % to 100 % and have a range from 0 to 1. Henseler et al. [85] proposed a general recommendation for acceptable R-squared, where 0.75, 0.50, and 0.25 are categorized as significant, moderate, and weak, respectively. Thus, a value of 0.25< r < 0.5 is typically regarded as a low impact size, a value of 0.5< r < 0.75 as a modest outcome size, and a value of R-squared value r > 0.75 as a large effect size. As the R^2^ score rises or approaches 1, the model's importance rises. The study established a second-degree polynomial equation for CT, SR, and MV using various input variables such as SS, FR, and DC. Eqs. (10), (11), (12), (13), (14), (15)) give equations for MV, CT and SR based on the findings of investigations. The R^2^ and S values for Eqs (10), (11), (12), (13), (14), (15)) are shown in Table 10. Eq. (10) outlines the link between SR, SS, and FR. The measured experimental data values are statistically fitted to the developed regression equation as similarly stated by Venkatesan et al. [86]. The estimated SR model's R^2^ value of 0.9905 is close to unity, indicating that no external influences were used to modify the fitted model. The SR has a significant influence on the DC and SS, as indicated by the R^2^ value of 0.8714 in Eq. (11). Moreover, Eq. (12) shows an R^2^ value of 0.9556, indicating a considerable influence for turning AISI 1525 steel using the SS and DC as the input variables. For the specified input process variables, the equation satisfies the statistical model well, with a good variation of the output response (CT). The CT regression analysis (FR and DC) is approximately at the moderately acceptable level of R^2^. In Eq. (13), the R^2^ value of 0.7527 indicates that the CT influences the FR and DC. In Eq. (14), an R^2^ value of 0.6973 indicates that machine vibration has a moderate impact on FR and SS. Finally, with an R^2^ value of 0.8524 from Eq. (15), the machine vibration model equation for SS and DC was generated. This demonstrates that the model is statistically sound. Moreover, ‘S' represents the difference between the fitted and raw values. The response is more precisely reflected in the equation as S decreases. The results show that, for a given range of SR, machine vibration, and CT, the model's prediction is correct. Using this data, one can determine the cutting variables for machine vibration, SR, and CT.

(10) $$SR\left(\mu m\right)=4.33-0.000605SS-0.95FR+0.000001SS^{2}-2.67FR^{2}+0.00692SS*FR$$

(11) $$SR\left(\mu m\right)=2.48+0.00162SS+2.84DC+0.000001SS^{2}-1.15DC^{2}-0.00119DC*SS$$

(12) $$CT\left({}^{\circ}C\right)=481.7+0.249SS-952DC-0.000121SS^{2}+481.6DC^{2}-0.0383DC*SS$$

(13) $$CT\left({}^{\circ}C\right)=393+1499FR-835DC-1720FR^{2}+482DC^{2}-912FR*DC$$

(14) $$MV\left(m/s^{2}\right)=10.9-0.0179SS-73.7FR+0.000018SS^{2}+231FR^{2}+0.0256SS*FR$$

(15) $$MV\left(m/s^{2}\right)=9.7-0.0041SS-11.7DC+0.000018SS^{2}+7.07DC^{2}-0.00995SS*DC$$

**Table 10:** Corresponding S and R^2^ values.

Eq.No.	S	$R^{2}$
10	0.0341802	0.9905
11	0.1258740	0.8714
12	6.9714800	0.9556
13	16.457900	0.7527
14	1.1080400	0.6973
15	0.7737390	0.8524

### 3.4 Assessment of surface and contour plots

**Fig. 14:**
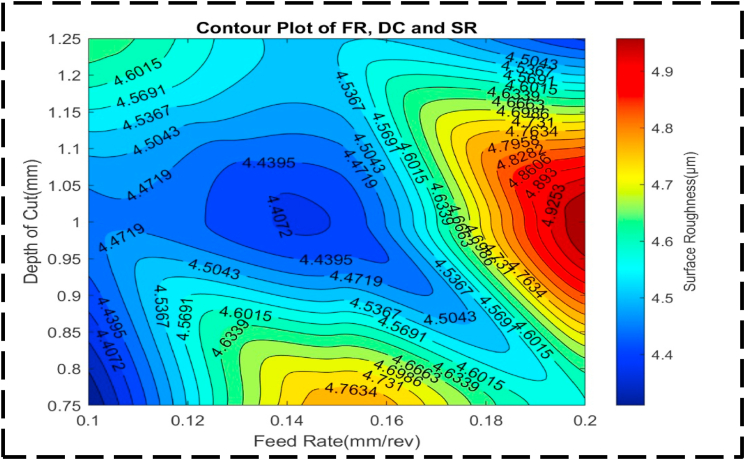
The contour diagram of FR, DC, and SR.

**Fig. 15:**
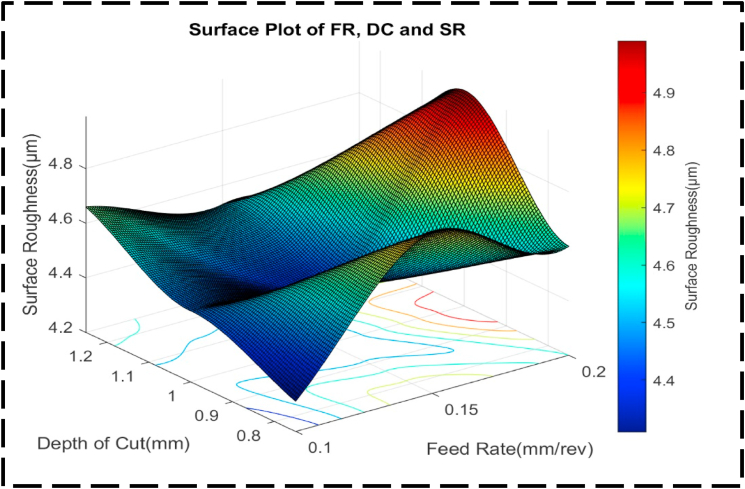
The surface pattern of DC, FR, and SR.

Fig. 14 presents an analysis of the relationship between the nondependent variables, FR and DC, and their influence on the response variable, SR. Two distinct peaks can be observed, representing the combinations of FR and DC that yield the highest SR. The first peak, with an SR of approximately 5.0 μm, occurs when the FR ranges from 0.19 to 0.2 mm/rev and the DC ranges from 0.95 to 1.05 mm. The second peak, with an SR of about 4.75 μm, is achieved with an FR ranging from 0.14 to 0.16 mm/rev and a DC ranging from 0.75 to 0.78 mm. Conversely, a valley in the plot indicates a lower SR. This peak is obtained when the FR ranges from 0.14 to 0.15 mm/rev, and the DC ranges from 1.00 to 1.04 mm. The contour lines in the plot are more firmly separated than parallel, and they exhibit curved shapes. This implies that the DC and FR interact with one another to have a combined effect on the SR greater than the effect of each factor operating independently [87,88]. The non-linear and concentric nature of the contour lines indicates that adjustments to both FR and DC are necessary to effectively control and optimize SR. The surface plot depicted in Fig. 15 exhibits an irregular concave shape, indicating a non-linear relationship between the FR, DC, and SR. The desired optimal minimum value for SR is approximately 4.3 μm. It is achieved when the FR is set at 0.11 mm/rev and the DC is set at 0.8 mm. Notably, the plot demonstrates asymmetry, suggesting that SR is not equally responsive to changes in the FR and DC, regardless of the direction of adjustment. This asymmetry implies that adjustments in either parameter will not have a comparable impact on SR.

**Fig. 16:**
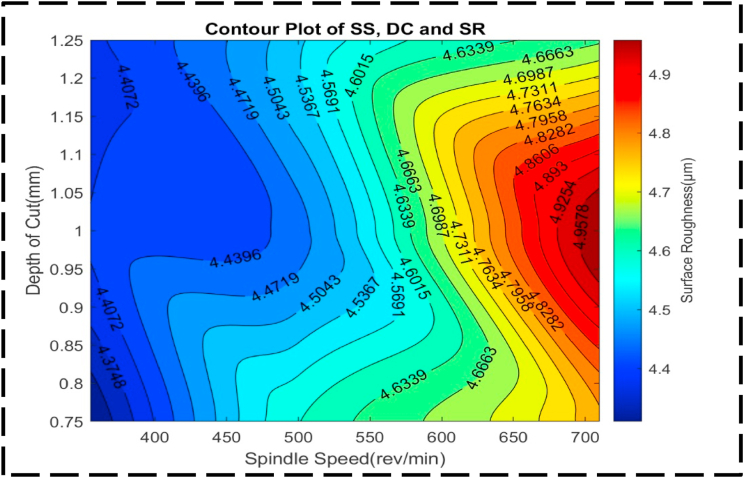
The contour diagram of DC, SS, and SR.

**Fig. 17:**
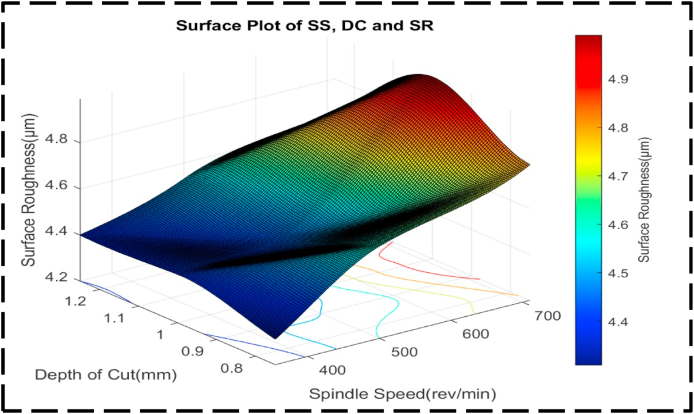
The surface pattern of DC, SS, and SR.

Fig. 16 shows how the response variable, SR, is affected by the nondependent variables, DC and SS. Notably, the plot reveals a distinct peak representing high SR and a corresponding valley representing low SR. The peak, characterized by an SR of approximately 5.0 μm, is achieved when the SS ranges from 700 to 710 rev/min, and the DC varies from 0.9 to 1.05 mm. Conversely, the valley, reflecting an SR of about 4.3 μm, corresponds to an SS range of 350–400 rev/min, and a DC ranging from 0.75 to 0.80 mm. Examining the contour lines within the plot, displayed were curved shapes and are more concentrically set apart rather than parallel. This suggests that the interaction between SS and DC has a significant influence on altering the SR, surpassing the individual effects of each factor. This was similarly reported by Refs. [89,90]. The non-linear and concentric nature of the contour lines highlights the importance of considering the combined impact of both variables when optimizing SR. Fig. 17 showcases a surface plot with an irregular convex shape, representing the relationship between the DC, SS, and SR. This shape indicates a non-linear association among these variables. Specifically, it reveals that as the SR, SS and DC increase, the SR experiences an increase, but at a decreasing rate. In other words, the SR becomes a little sensitive to the variation in DC and SS as their values increase. The plot indicates a minimum SR of 4.3 μm, which corresponds to an optimal DC of 0.8 mm and an SS of 350 rev/min. In this region, the SR is highly responsive to variations in the DC and SS, as indicated by the relatively steep slope. Consequently, achieving significant improvements in SR may require substantial adjustments in both the DC and SS. The irregular convex shape of the plot emphasizes the non-linear nature of the interaction between the DC, SS, and SR. It suggests that careful optimization of these parameters is crucial to achieving the desired SR, as the rate of change in SR varies across different regions of the plot as also reported in the work of Mia and Dhar; Davoodi and Tazehkandi [91,92].

**Fig. 18:**
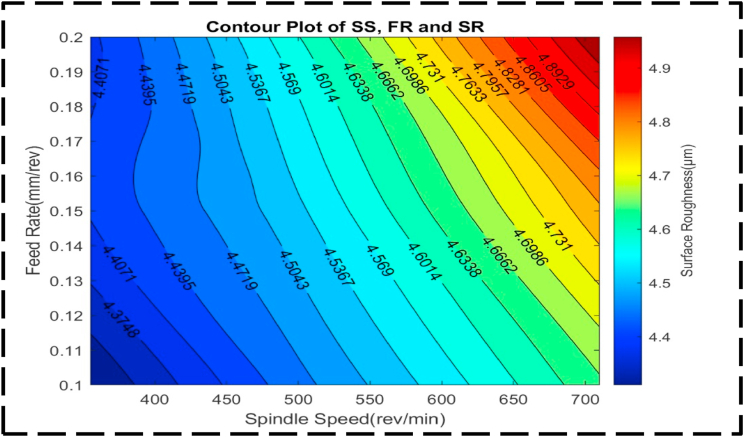
The contour diagram of SS, FR, and SR.

**Fig. 19:**
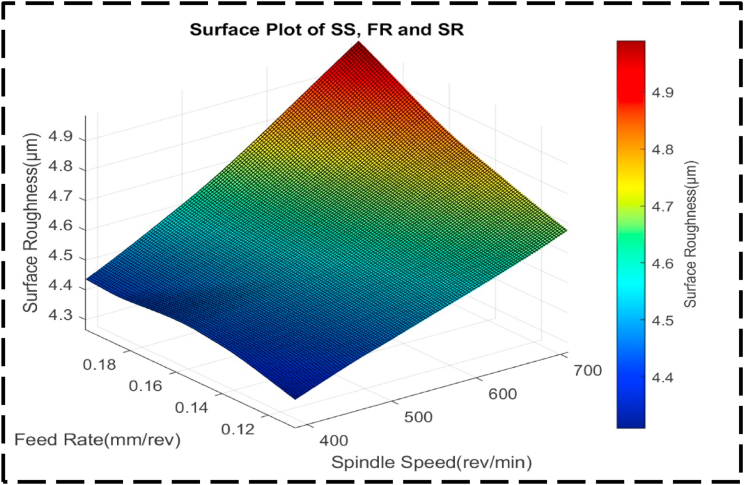
The surface pattern of FR, SS, and SR.

**Fig. 20:**
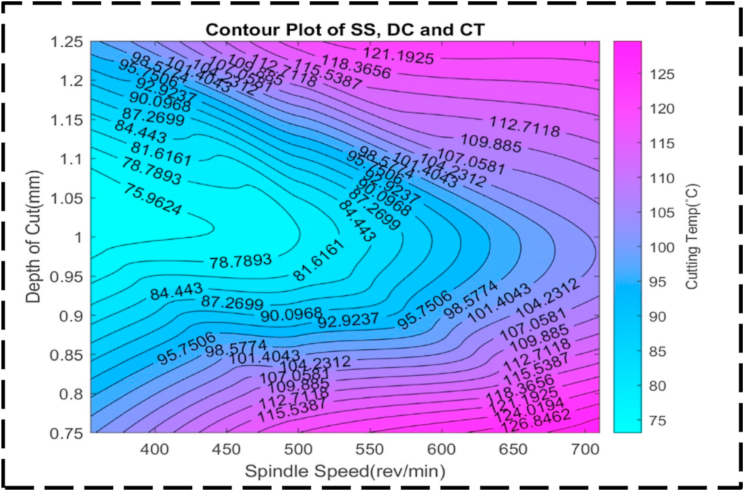
The contour diagram of SS, DC, and CT.

In Fig. 18, the response variable is SR, while the independent variables are SS and FR. The contour lines within the plot display a consistent parallel and linear orientation throughout. This observation indicates that changes in SS and FR occur at a constant rate and act independently to influence SR, with minimal interaction between the two variables [89,93,94]. The parallel and linear nature of the contour lines suggests that adjustments in SS and FR can be made separately and without significant consideration of their combined effect on SR. The regular form of the surface plot shown in Fig. 19 suggests that the SR, SS and FR have a linear relationship. Plotting demonstrates that a minimum SR value of 4.3 μm can be attained by setting the SS at 390 rev/min and the FR at 0.12 mm/rev. The extremely steep slope of the curve indicates that the SR is very sensitive to variations in the SS and FR in this particular region. This sensitivity suggests that large changes in SR would necessitate large changes in the SS and FR. The linear link between the SS, FR and SR is shown by the surface plot's regular form, which highlights how crucial exact control over these factors is to be achieving the appropriate SR level. The link between the independent variables, DC and SS, and their impact on the response variable, CT, is thoroughly examined in Fig. 20. Within the plot, a distinct valley can be observed, indicating the combined effect of DC ranging from 1.00 to 1.05 mm and SS varying from 350 to 450 rev/min. Notably, the highest levels of CT are associated with DC values between 1.20 and 1.25 mm, paired with SS ranging from 600 to 700 rev/min. Additionally, CT reaches peak values with alternative DC and SS combinations of 0.75–0.8 mm and 600–700 rev/min, respectively. The contour lines exhibited in this plot demonstrate a non-linear trend, indicating that changes in the FR and SS occur at varying rates within these regions. This implies that the CT is significantly influenced by the interaction between DC and SS, which is greater than the effect of each element acting separately [74,87].

**Fig. 21:**
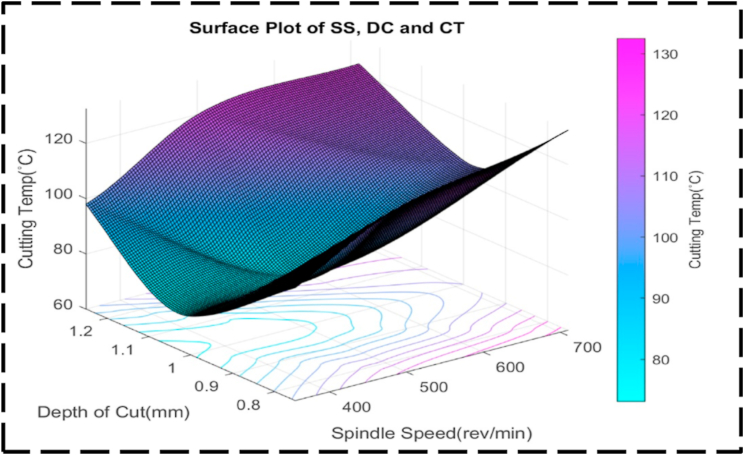
The surface pattern of DC, SS, and CT.

**Fig. 22:**
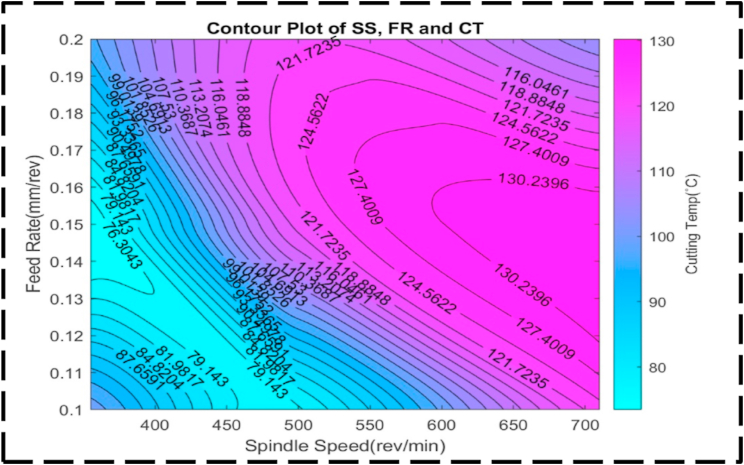
The contour diagram of SS, FR, and CT.

**Fig. 23:**
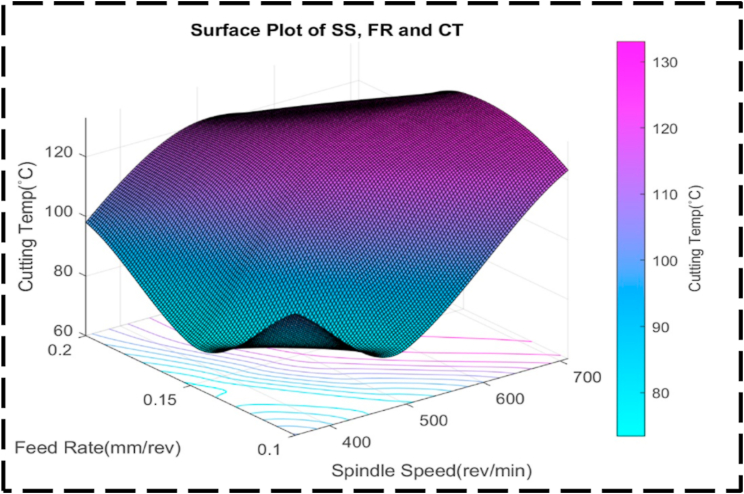
The surface pattern of FR, SS, and CT.

**Fig. 24:**
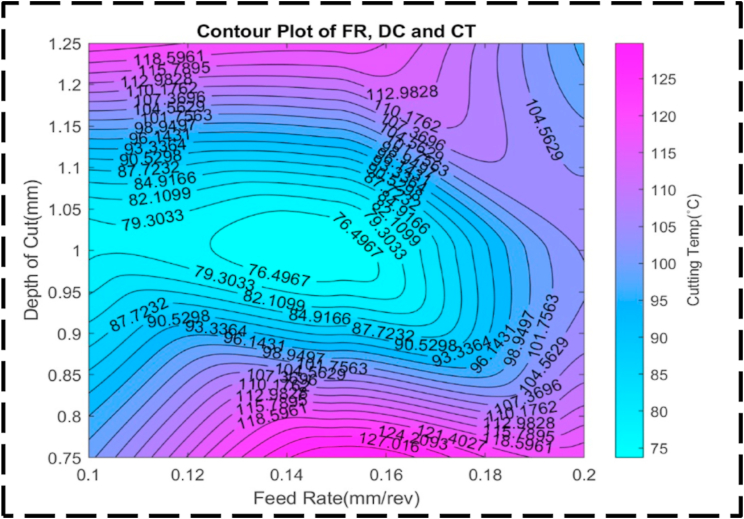
The contour diagram of DC, FR, and CT.

**Fig. 25:**
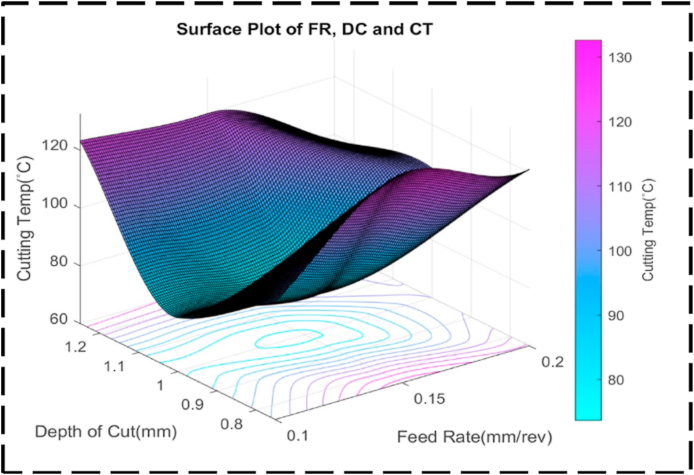
The surface pattern of DC, FR, and CT.

There is less of an interaction between the DC, SS, and CT as seen by the uneven form of the surface plot shown in Fig. 21. This anomaly implies that there fails to be a linear relationship between these factors. Presumably, symmetry in the plot means that, irrespective of the direction of correction, the CT is also susceptible to changes in the DC and SS [95]. This symmetry suggests that changes in any of the parameters can have a similar effect on the transformation time. Fig. 22 illustrates the relationship between the nondependent variables, SS and FR, and their impact on the response variable, CT. The plot highlights that the highest CT, exceeding 130 °C, is achieved when SS ranges from 600 to 700 rev/min, and FR varies between 0.12 and 0.17 mm/rev. This combination represents the peak of the plot, indicating the most extreme CT values. The contour lines observed in the plot display linearity between the peak and minor valley regions. This linearity suggests that changes in SS and FR occur at a constant rate within this specific range. The contour lines in this region demonstrate consistent and predictable variations in CT based on adjustments to SS and FR. At higher CTs, the contour lines become concentric, indicating that there is a significant interaction between SS and FR. The concentric contour lines indicate that, within this range, the effects of each variable on CT appear to be reliant upon one another. The comparatively regular form of the surface plot shown in Fig. 23 suggests a linear relationship between the SS, FR, and CT. When the SS is set at 350 rev/min and the FR is set at 0.14 mm/rev, the CT can be optimized to a minimum value of about 70 °C. Interestingly, the figure exhibits symmetry, indicating that, independent of the direction of adjustment, the CT is appropriately responsive to changes in the SS and FR. This symmetry implies that adjustments in either parameter can have a comparable impact on the CT [96,97]. Fig. 24 presents a comprehensive analysis of the intricate association between CT, the dependent variable, and the independent variables of FR and DC. Notably, a distinct valley is discernible, indicating a specific range of FR (0.12–0.16 mm/rev) and DC (0.95–1.05 mm) where CT reaches their minimum levels, measuring approximately 75 °C. Further examination of the contour lines within the plot reveals a noteworthy characteristic: their overall curvature. This curvature signifies that the rate of change in both FR and DC varies non-linearly between the contour lines. Such behavior emphasizes the interactive nature of these cutting parameters, as their combined influence significantly affects CT. It is worth noting that the contour lines illustrate that their collective impact on CT exceeds what could have been predicted by considering each factor in isolation. The profile of this surface plot in Fig. 25 is asymmetrical, indicating a diminishing correlation between the CT, DC, and FR [98]. This indicated that the relationship between the feed rate, DC, and the cutting temperature is non-linear. The required optimal least cutting temperature is approximately 68 °C, and the combinatorial values for this are 0.15 mm/rev and 1.2 mm. The symmetric nature of the plot suggests the sensitivity of the cutting temperature to variations in the feed rate and DC in either direction.

**Fig. 26:**
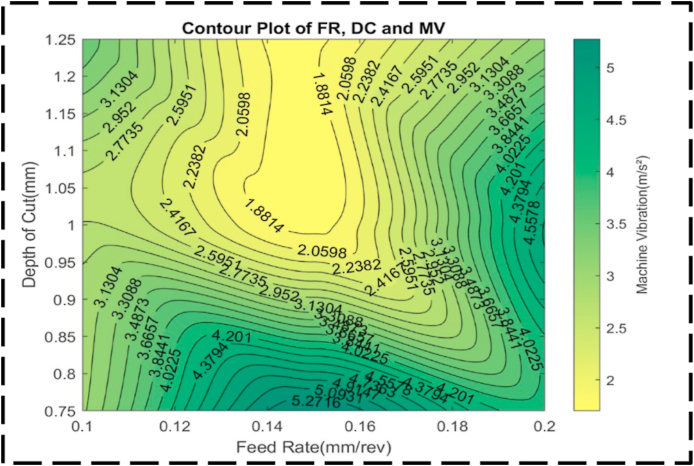
The contour diagram of DC, FR, and machine vibration.

**Fig. 27:**
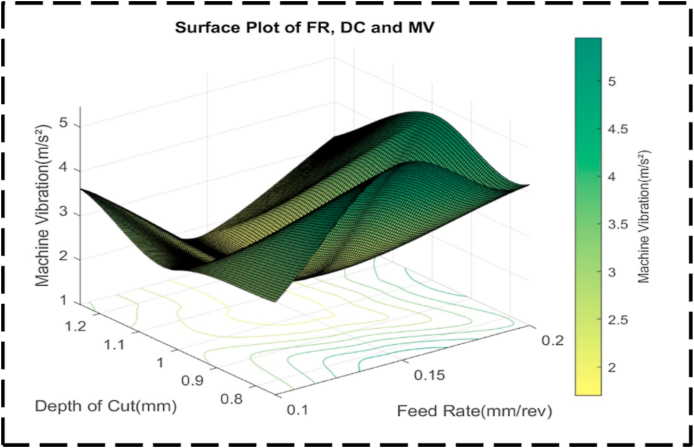
The surface pattern of DC, FR, and machine vibration.

Analyzing the contour lines within the plot in Fig. 26 reveals an interesting characteristic: their generally curved nature. This curvature signifies that the variation in FR and DC takes at an inconsistent proportion between the contour lines. Such behavior highlights the interaction between these cutting parameters, as they collectively influence machine vibrations. The contour lines demonstrate that their combined effect alters machine vibrations to a great extent. Fig. 27 illustrates an irregular surface plot, indicating a non-linear relationship between the FR, DC, and machine vibration. The optimal minimum value for machine vibration, approximately 1.5 m/s^2^, is achieved by setting the FR to 0.15 mm/rev and the DC to 1.05 mm. It is important to note that the plot demonstrates asymmetry, suggesting that machine vibration does not equally respond to changes in the FR and DC, regardless of the direction of adjustment. This indicates that modifications in either parameter cannot have an equivalent impact on machine vibration.

**Fig. 28:**
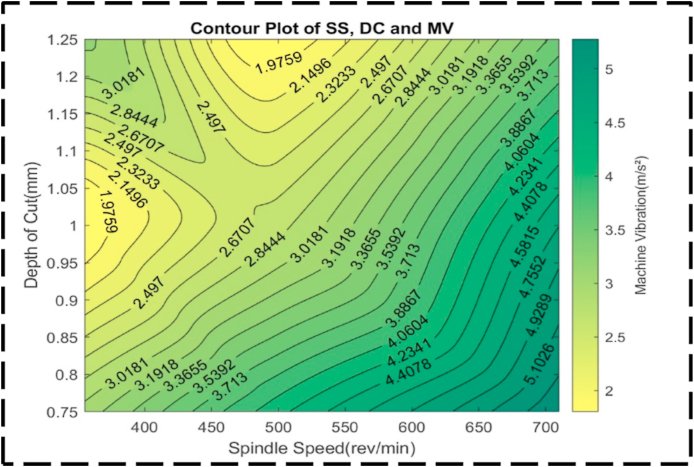
The contour diagram of DC, SS, and machine vibration.

**Fig. 29:**
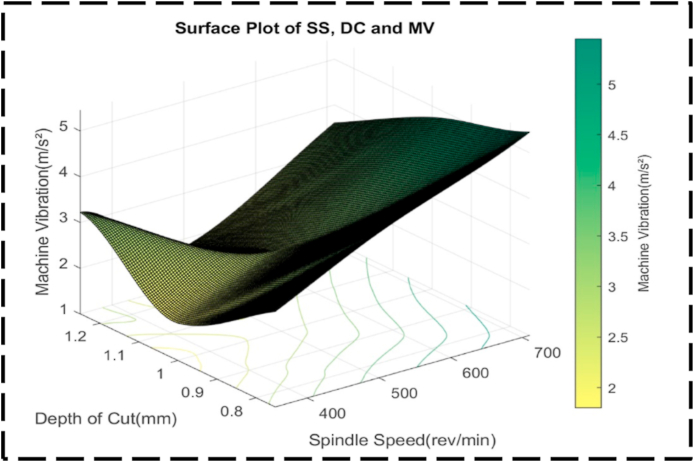
The surface pattern of DC, SS, and machine vibration.

**Fig. 30:**
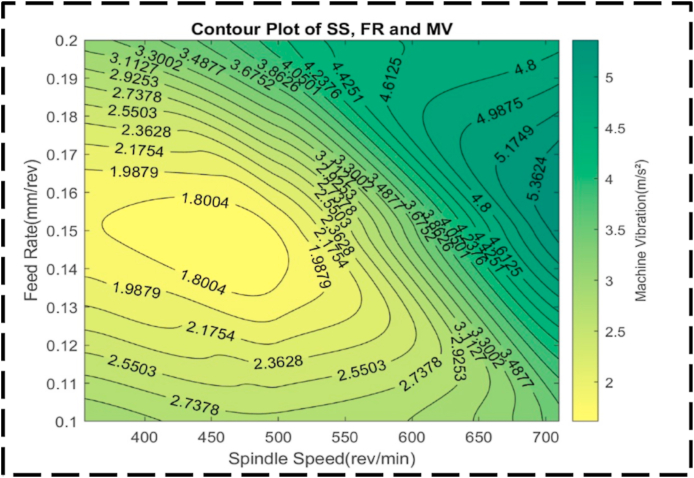
The contour diagram of FR, SS, and machine vibration.

Fig. 28 presents a comprehensive examination of the relationship between machine vibrations, the response variable, and the independent variables of SS and DC. Notably, a distinct peak is not observed in this plot; however, there are clear trends that can be discerned. As SS increases, machine vibrations gradually increase, whereas an increase in DC is associated with a decrease in machine vibrations. Analyzing the contour lines within the plot reveals intriguing characteristics. At lower machine vibrations, the contour lines exhibit a generally curved nature, indicating non-constant changes in the cutting parameters. Conversely, at higher machine vibrations, the contour lines demonstrate a more linear nature, suggesting more consistent changes in the cutting parameters. This observation indicates that at higher SS, the alterations in the cutting parameters occur at a relatively constant rate, while at greater depths of cut, the changes are non-constant [99]. The contour lines indicate varying degrees of interaction between the cutting parameters based on the level of machine vibrations. At lower machine vibrations, there is a higher level of interaction between the cutting parameters, whereas, at higher machine vibrations, the interactions are comparatively less. Fig. 29 depicts an irregular surface plot, which effectively conveys the existence of a non-linear correlation between SS, DC, and machine vibration. Remarkably, the investigation reveals that the most favorable outcome in terms of minimal machine vibration, approximately 1.5 m/s^2^, can be attained by configuring the SS to 350 rev/min and the DC to 1.00 mm. Significantly, it is imperative to acknowledge the symmetrical nature of the plot, suggesting that alterations in both SS and DC yield a comparable effect on machine vibration, regardless of the direction of adjustment. This observation implies that adjustments made to either parameter will exert an equivalent influence on machine vibration. Fig. 30 offers a comprehensive analysis of the relationship between the nondependent variables, SS and FR, and their impact on the response variable, machine vibrations.

**Fig. 31:**
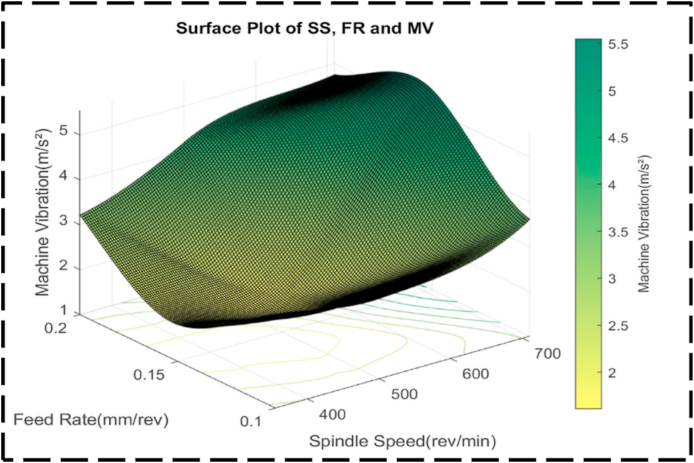
The surface pattern of FR, SS, and machine vibration.

Within the plot, it is evident that the highest machine vibrations, exceeding 5.0 m/s^2^, are attained at approximately 700 rev/min SS, with FRs ranging between 0.15 and 0.18 mm/rev. This combination represents a peak in the plot, indicating the most extreme machine vibration values. On the other hand, the desirable target for machine vibrations is approximately 1.5 m/s^2^, achieved at SS between 400 and 500 rev/min, and FR ranging from 0.135 to 0.16 mm/rev. These parameters result in reduced machine vibrations, indicating an optimized machining condition [[100], [101], [102]]. Examining the contour lines within the plot, linearity is observed between the peak and minor valley regions. This linearity suggests that changes in SS and FR occur at a constant proportion within this specific range. The contour lines demonstrate consistent and predictable variations in machine vibrations based on adjustments to SS and FR in this region. At the highest and lowest machine vibrations, the contour lines become concentric, indicating a significant interaction between SS and FR. This suggests that in these ranges, the effects of each variable on machine vibrations are dependent on one another, as evidenced by the concentric contour lines. The provided visual representation (Fig. 31) showcases a surface plot characterized by an irregular convex shape, effectively illustrating the intricate interplay between the FR, SS, and machine vibration. This shape serves as a visual indicator of the non-linear relationship existing among these variables. Specifically, the plot demonstrates that as the FR and SS increase, there is an associated increase in machine vibration. At lower values, the machine vibration exhibits heightened responsiveness to variations in the FR and SS, as evidenced by the relatively steep slope. Conversely, the shallow slope observed at significantly higher machine vibrations signifies a diminished sensitivity to adjustments in these parameters.

## 4 Conclusion

In this study, the effectiveness of watermelon oil was tested in comparison with conventional mineral oil during the turning of AISI 1525 steel with a tungsten carbide tool under the MQL strategy. The following results were drawn from the study.

1. The best result was achieved by watermelon lubricant yielding a 48 % reduction in surface roughness, a 4 % decrease in cutting temperature, and a 91 % reduction in machine vibration rates as compared with mineral lubricant. This indicated the possibility of the reduction of wear, friction, and superior corrosion resistance of the workpiece on which watermelon seed oil was applied. More so, the longevity of tool life and reduction in the wear rate of the tool is guaranteed with the low surface roughness of the workpiece.
2. Spindle speed at 710 rev/min, feed rate at 0.10 mm/rev, and cutting depth at 1.00 mm are the optimum and safest combination values for reducing surface roughness (SR), machine vibration, and cutting temperature for watermelon seed oil due to specific properties such high viscosity, superior lubricity, reasonable molecular weight.
3. Analysis of variance for GRG has confirmed that depth of cut is the major contributor to SR, cutting temperature, and machine vibration.
4. Finer grain sizes were observed at 70 mm diameter, with the predominant presence of pearlites. Hardness was also optimal at 70 mm diameter. These characteristics indicated the possibility of superior grain boundaries, higher deformation resistance, and impediment in dislocation movement at this diameter.

For a more extensive analysis, full factorial experimentation may be used to quantify the statistical analysis. Multiple objective optimizations with a broader scope may also be established.

## Author contribution statement

Stephen Akinwale Akinlabi: Writing – original draft, Visualization, Validation, Methodology, Funding acquisition, Data curation, Conceptualization. Tien-Chien Jen: Writing – review & editing, Writing – original draft, Supervision, Software, Investigation, Funding acquisition, Data curation, Conceptualization. Godwin Itopa Akande: Writing – review & editing, Writing – original draft, Data curation, Conceptualization. David Abimbola Fadare: Writing – review & editing, Supervision, Conceptualization. Esther Titilayo Akinlabi: Writing – original draft, Validation, Resources, Funding acquisition, Formal analysis, Data curation, Conceptualization. Rasaq Adebayo Kazeem: Writing – review & editing, Validation, Software, Investigation, Data curation, Conceptualization

## Funding statement

No specific grant was received from any funding agencies by the researchers.

## Data availability statement

Data included in article/supp. material/referenced in article.

## Additional information

This paper does not contain additional information.

## Declaration of competing interest

The authors declare that they have no known competing financial interests or personal relationships that could have appeared to influence the work reported in this paper.
